# An analytic perspective of a mixed methods study during humanitarian crises in South Sudan: translating facility- and community-based newborn guidelines into practice

**DOI:** 10.1186/s13031-021-00339-8

**Published:** 2021-01-12

**Authors:** Samira Sami, Ribka Amsalu, Alexander Dimiti, Debra Jackson, Kemish Kenneth, Solomon Kenyi, Janet Meyers, Luke C. Mullany, Elaine Scudder, Barbara Tomczyk, Kate Kerber

**Affiliations:** 1 Center for Humanitarian Health, Department of International Health, Johns Hopkins Bloomberg School of Public Health, 615 N. Wolfe Street, Baltimore, MD 21205 USA; 2 Save the Children, 2275 Sutter Street, San Francisco, CA 94115 USA; 3 Ministry of Health Republic of South Sudan, P.O.Box 336, Juba, South Sudan; 4 UNICEF/University of the Western Cape, 3 UN Plaza, New York, NY 10017 USA; 5 UNICEF, South Sudan, Totto Chan Compound, P.O.Box 45, Juba, Republic of South Sudan; 6 International Medical Corps. Tong ping Area block 3b, Juba, South Sudan; 7 Save the Children, 899 North Capitol Street NW, Suite 900, Washington, DC 20002 USA; 8 Department of International Health, Johns Hopkins Bloomberg School of Public Health, 615 N, Wolfe Street, W5009C, Baltimore, MD 21205 USA; 9 Center for Global Health, US Centers for Disease Control and Prevention, 1600 Clifton Rd, Bldg 21 Rm 9208 MS D-69, Atlanta, GA 30329 USA

**Keywords:** Newborn health, South Sudan, Conflict, Community, Facility, Health system, Displaced populations, Guideline translation

## Abstract

**Background:**

In South Sudan, the civil war in 2016 led to mass displacement in Juba that rapidly spread to other regions of the country. Access to health care was limited because of attacks against health facilities and workers and pregnant women and newborns were among the most vulnerable. Translation of newborn guidelines into public health practice, particularly during periods of on-going violence, are not well studied during humanitarian emergencies. During 2016 to 2017, we assessed the delivery of a package of community- and facility-based newborn health interventions in displaced person camps to understand implementation outcomes. This case analysis describes the challenges encountered and mitigating strategies employed during the conduct of an original research study.

**Discussion:**

Challenges unique to conducting research in South Sudan included violent attacks against humanitarian aid workers that required research partners to modify study plans on an ongoing basis to ensure staff and patient safety. South Sudan faced devastating cholera and measles outbreaks that shifted programmatic priorities. Costs associated with traveling study staff and transporting equipment kept rising due to hyperinflation and, after the July 2016 violence, the study team was unable to convene in Juba for some months to conduct refresher trainings or monitor data collection. Strategies used to address these challenges were: collaborating with non-research partners to identify operational solutions; maintaining a locally-based study team; maintaining flexible budgets and timelines; using mobile data collection to conduct timely data entry and remote quality checks; and utilizing a cascade approach for training field staff.

**Conclusions:**

The case analysis provides lessons that are applicable to other humanitarian settings including the need for flexible research methods, budgets and timelines; innovative training and supervision; and a local research team with careful consideration of sociopolitical factors that impact their access and safety. Engagement of national and local stakeholders can ensure health services and data collection continue and findings translate to public health action, even in contexts facing severe and unpredictable insecurity.

## Background

### Humanitarian context

After a period of relative peace following independence in 2011, South Sudan experienced acute and sudden conflict in December 2013. The situation worsened in 2016 and led to mass displacement in Juba. This rapidly spread to other regions of the country such as the Upper Nile and areas with relative peace and stability [1]. Fragmentation of political groups and longstanding intercommunal tensions intensified and the number of persons seeking refuge in United Nations (UN) protection of civilian (POC) camps substantially increased [2]. By the end of 2017, about 1.9 million people were estimated to be internally displaced in a country of 10.8 million people [3, 4]. The UN POC camps in Juba and Malakal predominately housed displaced women and children who were from the Nuer and Shilluk tribes [1]. Additionally there were over 260,000 refugees from Sudan mostly residing in Upper Nile [5]. Decades of conflict, insecurity, mass displacement, and limited government investment weakened the health system in South Sudan, leaving behind long-term negative public health impact. The country faced a neonatal morality rate (NMR) of 39 per 1000 live births and stillbirth rate of 30 per 1000 total births, one of the highest in Africa [6–8].

### Research study

During 2016 to 2017, we undertook a study in four displaced person camps located in Upper Nile State (Maban and Malakal Counties) and Juba Central Equatoria State to assess the implementation of a package of newborn health interventions at the community and facility levels during a protracted humanitarian emergency [9–11]. Our study objectives were to (1) examine change in knowledge and attitudes of community- and facility-based health workers toward newborn health interventions (i.e. acceptability), (2) assess change in newborn care practices during childbirth and the immediate postnatal period (i.e. adoption), and (3) explore health system factors that influence implementation. To address the first two objectives, we designed a quasi-experimental pre-post study with clinical observations of delivery and postnatal care practices, structured exit interviews with recently delivered women, and semi-structured interviews with health workers (see Table 1). To address the third objective, we used a mixed methods approach with in-depth interviews, focus group discussions, health facility checklists, and health worker time-use observations at multiple time points during implementation.

**Table 1:** Study data collection methods and sample size, April 2016—January 2017

**Phase 1: Baseline** **April – June 2016**	Health facility assessment	5 health facilities
	Time use observation	1163 observations
	Clinical observation and exit interview	Hospital: 159 mother-newborn pairs PHCC: 201 mother-newborn pairs
	In-depth interview	17 health workers
	Self-administered knowledge questionnaire	127 health workers
**Phase 2: Midline** **July – November 2016**	Health facility assessment	5 health facilities
	In-depth interview	16 health workers 7 program managers
	Focus group discussion	12 facility health worker groups 8 community health worker groups
	Supply consumption	5 health facilities 3 community health program sites
**Phase 3: Endline** **November 2016 – January 2017**	Health facility assessment	5 health facilities
	Time use observation	565 observations
	Clinical observation and exit interview	Hospital: 106 mother-newborn pairs PHCC: 127 mother-newborn pairs
	In-depth interview	10 health workers 4 program managers
	Focus group discussion	3 facility health worker groups 2 community health worker groups

Of the four displaced person camps, two were refugee camps in Maban County (Gendrassa and Kaya) and two were internally displaced persons (IDP) camps in Malakal County (Malakal POC) and Juba County (Juba POC), with populations ranging from 17,000 to 40,000 displaced persons [3, 5]. In June 2016, International Medical Corps (IMC), an international humanitarian organization, implemented the study intervention in the camps, including: clinical training and ongoing supportive supervision of community- and facility- based health workers; distribution of newborn medical commodities at the community, primary care, and hospital level; and a strategic planning workshop for senior managers to prioritize programmatic considerations. Facility-based newborn interventions were implemented in a primary care facility in each camp and a hospital in Juba POC. Community-based newborn interventions were integrated in the community health program of all camps.

The study sites were prone to sudden conflict and attack because of the political and socioeconomic circumstances in and around the camps. A month prior to the study, a violent attack in Malakal POC led to civilian and health worker deaths and one of the study health facilities was burnt down [12]. During the study in July 2016, a maternity ward in Juba POC was shelled and tensions between refugee and host populations in Maban led to fighting and displacement [13]. Humanitarian agencies, including IMC, were forced to frequently suspend operations and evacuate non-local staff. The ongoing crisis introduced unexpected changes to the study intervention and the quasi-experimental study design became susceptible to threats to internal validity. The study design shifted to a descriptive analysis of knowledge, attitudes, and practices for newborn care without comparison to a baseline [14].

The study findings indicated acceptability and adoption of newborn health interventions among facility- and community-based health workers were high after delivering a two-day simulation training and supplies. Knowledge of newborn danger signs and benefits of practices such as skin-to-skin contact and early breastfeeding initiation improved among health workers [10]. Improvements in knowledge, however, did not lead to adoption of interventions at the community level. Postnatal home visits in the first week of life, while new and acceptable to community health workers, were not sustained during periods of mass displacement because of the inability to locate households and limited staff available to manage competing priorities [11]. In facilities, partograph use for fetal monitoring, skin-to-skin contact, and postnatal monitoring of danger signs were the least commonly used practices at baseline, highlighting gaps in care for small and sick newborns [9]. Despite this, essential newborn practices such as thermal care (immediate drying and wrapping), infection prevention, and feeding support were high following the intervention. Addressing certain health system bottlenecks influenced implementation of study interventions, particularly: (1) leadership and governance to support comprehensive services, (2) health workforce for skilled care at birth, and (3) service delivery for small and sick newborns [11]. This case analysis describes the challenges encountered and mitigating strategies employed during the conduct of this research study.

## Discussion

### Scientific importance of research

Progress in reducing neonatal mortality is lagging behind improvements made in child survival after the first month of life, and South Sudan continues to have the highest NMR in Africa [15]. Evidence-based guidelines describe the most effective interventions to prevent and manage the main causes of newborn death [16]. However, countries with a high NMR have recently experienced a humanitarian emergency and the translation of newborn guidelines into public health practice during periods of on-going violence is not well understood or operationalized in these contexts [17].

During the inception phase, the study team drafted a dissemination plan to ensure findings would be shared with partners in South Sudan and the global community. Since then, the team held several workshops in Juba with the South Sudan Reproductive Health Technical Working Group to encourage uptake of findings. IMC program managers in Juba, Malakal, and Maban also received biweekly program updates, including supply stockouts, from the study supervisors in each site. The study led to the first national workshop on newborn health in South Sudan, co-hosted by the Ministry of Health and UNICEF, which set the stage for drafting an Every Newborn Action Plan (ENAP) for the country. South Sudan’s ENAP has led to the development of a newborn service package under the main health funding mechanism in the country. The National Community Health Strategy has also been revised to incorporate community-based newborn interventions.

At the global level, learnings were used beyond humanitarian settings to inform methodologies for measuring newborn care signal functions in other low resource settings and a newborn medical supply kit for community- and facility-based care [18, 19]. The findings of this research have been shared through the Newborn Health in Emergencies webpage hosted by Save the Children [20], blogs disseminated through the Healthy Newborn Network [20–22], a correspondence published in The Lancet [23], a public webinar as part of Save the Children’s Health and Nutrition Series [24], poster and oral presentations at several international conferences, and research articles in peer-reviewed journals [9–11]. Several policy and programmatic changes were also made at the global level such as development of a global roadmap to accelerate newborn health program scale-up in humanitarian settings and the Newborn Health Humanitarian Settings Field Guide that included an implementation toolkit [19, 25, 26].

### Strategies to address research challenges

#### Methodological issues

Study sites experienced frequent periods of conflict and insecurity, which posed several challenges for the study methodology including staff capacity and ethical concerns. In the design phase, facility-based newborn care practices were intended to be compared pre- and post-intervention. However, attacks against health facilities and workers led IMC to limit service delivery to ensure staff and patient safety. Maternity wards were also moved to alternative locations until destroyed facilities were rebuilt and newborn supplies were shifted for other purposes. Due to high turnover of health workers at health facilities, about half of the workers who were trained as part of the study intervention remained in the sites. This limited the study to a descriptive analysis.

While insecurity in South Sudan presented many sudden challenges, study co-investigators represented a diverse group of agencies, including non-governmental organizations (NGO), Ministry of Health, and academia, that offered creative strategies for adapting research methods in the constantly changing environment. Partnerships with non-research NGOs and UN agencies such as IMC, Save the Children, United Nations High Commissioner for Refugees (UNHCR), and UNICEF, who had substantial experience adapting clinical services during acute conflict, proved vital for identifying similar methods to sustain study operations. For instance, clinical observations for measuring newborn care practices in prior studies were conducted by research assistants with a clinical background such as nursing or midwifery, but this was not an option in a country facing extreme health workforce shortages. Instead, we worked closely with NGOs to identify strong candidates in the community and designed a data collection training to meet varying educational levels including tools and equipment for illiterate health workers. The training introduced basic clinical practices, such as partograph use, resuscitation, essential newborn care, and kangaroo mother care, that would be observed by researcher assistants. This required allocating additional funds to extend the data collection training from an 8 to a 15-day period.

#### High staff turnover

As ethnic tensions and insecurity rose, more than half of facility-based health workers who participated in the study intervention left their position. This included restricting movement of non-local staff in the evening hours and temporarily staffing maternity wards with traditional birth attendants (TBA). We worked closely with the donor to allocate additional funds for a second round of training for newly hired health workers. The study team also partnered with another UN agency, UNHCR, to integrate the content in upcoming trainings in Maban. When TBAs were hired to conduct deliveries in study facilities due to evacuation of non-local midwives, we needed to carefully consider expanding the study intervention and training to include TBAs. This became a critical aspect for understanding the feasibility of implementing newborn care in contexts that most represent conflict-affected settings. Inclusion of local community members in the study team also allowed the study to continue with data collection during periods of insecurity when others were unavailable. This limited research staff turnover and improved consistency in the application of data collection methods.

#### Ethical and safety concerns

Study operations were designed with a research coordinator in Juba and a supervisor plus four to five data collectors in each site. During times of episodic violence, the safety of local researchers was the primary concern of the study team and implementing partners. Frequent discussions about staff safety included what is the degree of additional risk, if any and how risks could be minimized. We recruited a field study team who were either from the community or lived in the camp to avoid potential ethical and safety concerns. This meant hiring staff who represented diverse ethnicities in South Sudan such as Shilluk, Dinka, and Nuer people. Because of the insecurity following the July 2016 crisis and armed groups targeting civilians based on ethnic lines, we no longer held joint data collection trainings in Juba or elsewhere for the study sites. Throughout the study, the team worked closely with security officers in partnering agencies to anticipate how and when research staff could access sites. This included adopting communication and transportation protocols used by NGO program staff to ensure the safety of the research team.

#### Remote monitoring of research activities

Shortly after the violence in Juba in July 2016, study co-investigators were unable to return to South Sudan to conduct trainings or monitor data collection as described in the original study protocol. Mobile data collection on tablets allowed data collectors to upload quantitative data every 24 to 48 h using wireless internet at the IMC field offices. When site supervisors and co-investigators were unable to visit study sites, they were able to conduct daily reviews of the data using the online database. Missing or erroneous data were reported immediately to the site supervisors. The close working relationship with IMC allowed us to identify practical strategies to ensure tablets were adequately maintained, charged, and safely stored in remote areas. When staff movement between facilities and IMC offices were restricted, additional tablets were purchased to reduce disruptions in data collection or uploads. Lastly, because of the targeting of ethnic minority groups and ongoing insecurity, staff were not allowed to convene in Juba. The study co-investigator, research coordinator and site supervisors met in Entebbe, Uganda for a one-week refresher training to overcome the travel restrictions that were imposed during the conflict. Supervisors then returned to their study sites and trained local data collectors. This cascade approach built the capacity of local researchers in qualitative and quantitative methods and allowed data collection to continue with remote support.

#### Budget implications

South Sudan presented numerous logistical challenges because of the ongoing conflict. While costly, equipment for the study intervention were transported in the country using plane because of the high risk for armed attacks along roads. Costs associated with transporting study staff and equipment kept rising due to hyperinflation of the local currency. Because of the high staff turnover, additional funding was needed for this and to extend the study timeline to re-train health workers and reorder additional job aids and training supplies.

#### Competing health priorities

In June 2016, South Sudan faced a cholera outbreak that shifted staffing and response priorities. As a result, program managers in the community and facility had limited capacity to maintain weekly supervision tasks related to the study interventions. Study supervisors were also often requested to support clinical supervision. With IMC input, the team developed a staffing plan so that each site had an adequate number of researchers to support their activities and avoid burdening program staff, which proved to be critical during the cholera outbreak and other strenuous moments on the health system. The engagement of MOH from the beginning was critical in the absorption of learning and using the research finding to inform the ENAP that was later developed for the country.

## Conclusions

Our case analysis provides specific lessons from the field including adaptable research methods, flexible budgets, innovative training and supervision of field researchers, and consideration of sociopolitical factors that affected the research team’s safety and access throughout training and data collection. Building diverse partnerships allowed for more informed decision-making starting from the design to dissemination phase. Careful consideration of different aspects of the study, including design, staff capacity, ethical concerns and logistics, lead to creative solutions for completing the study in the context of a humanitarian emergency. The study also produced substantial programmatic and policy changes, including a national level ENAP, because of the collaboration with NGOs, UN, and the Ministry of Health that led to widespread dissemination of the findings. Completion of this study was largely due to engaging community members as part of the study team who continued data collection in times of greatest insecurity knowing the outcomes would help their communities. Conducting much needed implementation research in conflict settings is rare due to the many challenges described in this paper. This case analysis provides important lessons for future research in these settings to assure quality services to highly disadvantaged populations.

## Data Availability

The datasets used and/or analyzed during the current study available from the corresponding author on reasonable request.

## Abbreviations

ENAP: Every Newborn Action Plan
IDP: Internally displaced person
IMC: International Medical Corps
POC: Protection of civilians
NGO: Non-governmental organization
NMR: Neonatal mortality rate
TBA: Traditional birth attendant
UN: United Nations
